# Efficacy of vitrectomy combined with and without cataract surgery for diabetic macular edema: one-year follow-up multi-center study in Japan

**DOI:** 10.1007/s00417-025-06845-2

**Published:** 2025-05-06

**Authors:** Yutaka Yamada, Yoshihiro Takamura, Kazuma Saito, Akira Minamoto, Gaku Ishigooka, Takashi Koto, Yuki Nakano, Eiko Tsuiki, Hiroto Terasaki, Miho Shimizu, Masayo Kimura, Yoshinori Mitamura, Reio Sekine, Yutaro Mizusawa, Takao Hirano, Tomoyuki Oyama, Takeshi Iwase, Fumiaki Higashijima, Hisashi Matsubara, Masaru Inatani

**Affiliations:** 1 Department of Ophthalmology, Faculty of Medical Sciences, University of Fukui, Yoshida, Japan; 2 Department of Ophthalmology, Gunma University Graduate School of Medicine, Maebashi, Japan; 3 Department of Ophthalmology and Visual Science, Hiroshima University, Hiroshima, Japan; 4 Department of Ophthalmology, Osaka Medical and Pharmaceutical University, Takatsuki, Japan; 5 Department of Ophthalmology, Kyorin University School of Medicine, Tokyo, Japan; 6 Department of Ophthalmology, Faculty of Medicine, Kagawa University, Miki, Japan; 7 Department of Ophthalmology and Visual Sciences, Graduate School of Biomedical Sciences, Nagasaki University, Nagasaki, Japan; 8 Department of Ophthalmology, Kagoshima University Graduate School of Medical and Dental Sciences, Kagoshima, Japan; 9 Sapporo City General Hospital Ophthalmology, Hokkaido, Japan; 10 Department of Ophthalmology and Visual Science, Nagoya City University Graduate School of Medical Sciences, Nagoya, Japan; 11 Department of Ophthalmology, Tokushima University Graduate School, Tokushima, Japan; 12 Department of Ophthalmology, St Marianna University School of Medicine, Kawasaki, Japan; 13 Department of Ophthalmology, Nara Medical University, Kashihara, Japan; 14 Department of Ophthalmology, Shinshu University School of Medicine, Matsumoto, Japan; 15 Department of Ophthalmology, National Defense Medical College, Tokorozawa, Japan; 16 Department of Ophthalmology, Akita University Graduate School of Medicine, Akita, Japan; 17 Department of Ophthalmology, Yamaguchi University School of Medicine, Ube, Japan; 18 Department of Ophthalmology, Mie University Graduate School of Medicine, Tsu, Japan

**Keywords:** Diabetic macular edema, Vitrectomy, Cataract surgery, Epiretinal membrane

## Abstract

**Purpose:**

To evaluate the efficacy of vitrectomy, with and without cataract surgery, for diabetic macular edema (DME) in Japan.

**Method:**

This retrospective study was conducted at 22 sites in Japan and enrolled patients who underwent vitrectomy either without (VIT group) or with (VIT + CS group) cataract surgery. Central retinal thickness (CRT) and best-corrected visual acuity (BCVA) were measured before surgery and at 1, 3, 6, and 12 months after surgery.

**Results:**

A total of 722 patients with DME (482 in the VIT + CS group and 240 in the VIT group) were enrolled. CRT significantly decreased after 1 month and continued thereafter in both groups. BCVA significantly improved at 1 month in the VIT + CS group and at 6 months in the VIT group. In both groups, regardless of epiretinal membrane removal, CRT and BCVA significantly improved, with no additional benefit from concomitant internal limiting membrane peeling. The change in BCVA was significantly correlated with the change in CRT during 6 months postoperatively in all patients and in the VIT group. Patients with worse preoperative visual acuity had a higher likelihood of improved BCVA at 6 and 12 months after surgery. No significant difference in BCVA was observed before and after surgery in patients with a preoperative visual acuity of 20/40 or better. However, in patients with a visual acuity of 20/50 or worse, BCVA significantly improved 1 month after surgery.

**Conclusion:**

Vitrectomy is anatomically and functionally effective for DME, and combined cataract surgery is beneficial in DME cases with cataracts. Patients with poor preoperative BCVA improved, while those with good vision maintained it. However, better preoperative vision increased the risk of postoperative deterioration, underscoring the need for careful evaluation of surgical indications.

**Key messages:**

***What is known***

- In vitrectomy for diabetic macular edema (DME), DRCR.net showed that 13–31% of patients experience decreased vision despite reduced edema, leaving the efficacy of vitrectomy uncertain.

***What is new***

- Our multicenter study demonstrated that vitrectomy with and without cataract surgery was effective in improving central retinal thickness and visual acuity in the patients with DME.
- Better preoperative visual acuity increased the risk of postoperative decline, and thus the need for careful evaluation of surgical indications.

## Introduction

Diabetic macular edema (DME) is the main cause of visual impairment in patients affected by diabetic retinopathy (DR) [1]. The pathophysiology of DME is primarily driven by long-term hyperglycemia, which leads to hypoxia, blood-retinal barrier breakdown, and vascular leakage. Currently, the intravitreal injection of anti-vascular endothelial growth factor (VEGF) agents is the gold standard and first-line treatment for DME [2]. Many clinical studies have demonstrated that anti-VEGF therapy is effective in improving macular swelling and visual acuity; however, some patients remain refractory to this treatment [3, 4]. Other treatment options include corticosteroid, laser therapy, and vitrectomy [1].

Vitrectomy, a surgical procedure that involves the removal of the vitreous gel from the eye, has been explored as a treatment option for DME, particularly in cases involving vitreomacular traction with an epiretinal membrane (ERM) or when other treatments fail to produce satisfactory results [5, 6]. The rationale for vitrectomy in DME is to relieve mechanical traction on the macula, improve oxygenation [7], and facilitate the removal of inflammatory factors contributing to macular edema [8]. Recently, vitrectomy has evolved to become safer and more efficient with the development of new technologies, including small-gauge instruments, wide-angle viewing systems, and safer dyes to visualize vitreous gels and internal limiting membranes (ILM) [9]. Although vitrectomy is not the first-line therapy for DME, its efficacy may be improving and should continue to be validated.

A prospective cohort study by DRCR.net found that over 80% of eyes with vitreomacular traction experienced decreased retinal thickness after vitrectomy. Additionally, nearly half of the patients showed improved visual acuity, while 13–31% experienced a decline in visual acuity [10]. Vitrectomy was performed with lens sparing, which may have resulted in vision loss due to cataract progression. In contrast, in Japan, except for younger patients with no obvious lens opacity and preserved accommodation, cataract surgery is performed in combination with vitrectomy in patients with DME and cataracts. Therefore, the effect of vitrectomy can be examined without the influence of postoperative cataract progression. We conducted a large-scale multicenter study in Japan to examine the effects of vitrectomy, with and without concomitant cataract surgery, on DME.

## Method

We collected data from 22 clinical centers throughout Japan. This study was performed in accordance with the Declaration of Helsinki and approved by the University of Fukui Institutional Review Board (IRB No. 20230129) and the ethics committees of the other participating hospitals. This study was conducted using an opt-out approach, where informed consent was not obtained from each participant. Participants were provided with information about the study, including its objectives and the use of their data, and were given the opportunity to decline participation. This study was registered with the University Hospital Medical Information Network Clinical Trials Registry (UMIN-CTR) of Japan (UMIN ID 000055641; date of access and registration: September 26, 2024). We retrospectively reviewed the medical records of patients with DME who underwent vitrectomy with (VIT + CS group) or without cataract surgery (VIT group). Data were collected for 12 months following the surgery.

Patients with type 2 diabetes and thickening of the macular center, defined as a central retinal thickness (CRT) of ≥ 300 μm in the central subfield based on spectral-domain optical coherence tomography (SD-OCT), were included in the study. Any vitreoretinal traction with ERM was removed if present.

From the medical records, the following information was obtained at the time of surgery: age, sex, HbA1c level, serum creatinine level, presence of dialysis or insulin therapy, grade of DR, history of laser surgery (PRP: panretinal photocoagulation, TRP: targeted retinal photocoagulation [11], and the number of injections of anti-VEGF drugs in the year before and after surgery. The vitrectomy incision size, presence of ERM peeling, ILM peeling, preparations used for staining, and the number of laser shots performed during surgery were also examined. Best-corrected visual acuity (BCVA) and CRT were examined preoperatively and at 1, 3, 6, and 12 months postoperatively.

The main exclusion criteria were (1) severe DME cases with CRT of > 1000 μm, (2) patients under 20 years of age, (3) focal/grid photocoagulation or panretinal photocoagulation within the previous 6 months, (4) lens-sparing vitrectomy, (5) active intraocular inflammation or infection in either eye, (6) neovascular glaucoma (NVG) before vitrectomy, (7) severe cataracts that prevented visualization of the OCT image, (8) history of vitrectomy and (9) vitreous hemorrhage or tractional retinal detachment. At 1, 3, 6, and 12 months postoperatively. All patients underwent complete ophthalmic examinations, including BCVA, intraocular pressure, fundus examination, and OCT. BCVA was converted to the logarithm of the minimum angle of resolution (logMAR) scale. Only one eye per participant was included in this study.

A standard pars plana vitrectomy was performed according to the surgeon’s usual routine. The general procedures included: (1) four pars plana sclerotomies using 23-, 25-, or 27-gauge microincision procedures with chandelier illumination, (2) removal of the vitreous gel along with peeling of the posterior hyaloid, (3) peeling of ERM deemed visually significant based on OCT images, and (4) ILM peeling and laser photocoagulation in the peripheral retina, performed at the surgeon's discretion. Phakic patients also underwent phacoemulsification and intraocular lens (IOL) implantation before vitrectomy. Postoperatively, all patients received similar routine medications: bromfenac sodium twice daily and betamethasone three times daily for 3 months.

Anti-VEGF drugs used for intravitreal injection included ranibizumab (Lucentis; Novoartis Pharma K.K., Tokyo, Japan) and aflibercept (Eylea; Bayer Yakuhin, Ltd., Tokyo, Japan) in 0.5 mg/0.05 mL and 2 mg/0.05 mL, respectively.

Statistical analyses were performed using JMP software (SAS Institute Inc., Tokyo, Japan). Data were presented as means ± standard deviations of the means. One-way repeated-measures analysis of variance with Greenhouse–Geisser correction was performed to examine whether the changes over time were statistically significant. Steel’s multiple comparison test was used to compare continuous variables within a group. We examined the differences between the groups using the Mann–Whitney U test as a nonparametric test. Simple regression analysis was used to examine the relationship between the change of CRT (ΔCRT) and BCVA (ΔBCVA). ΔBCVA for 6 months was calculated as BCVA at 6 months after surgery minus BCVA before surgery. Statistical significance was set at *p* < 0.05.

## Results

**Fig. 1:**
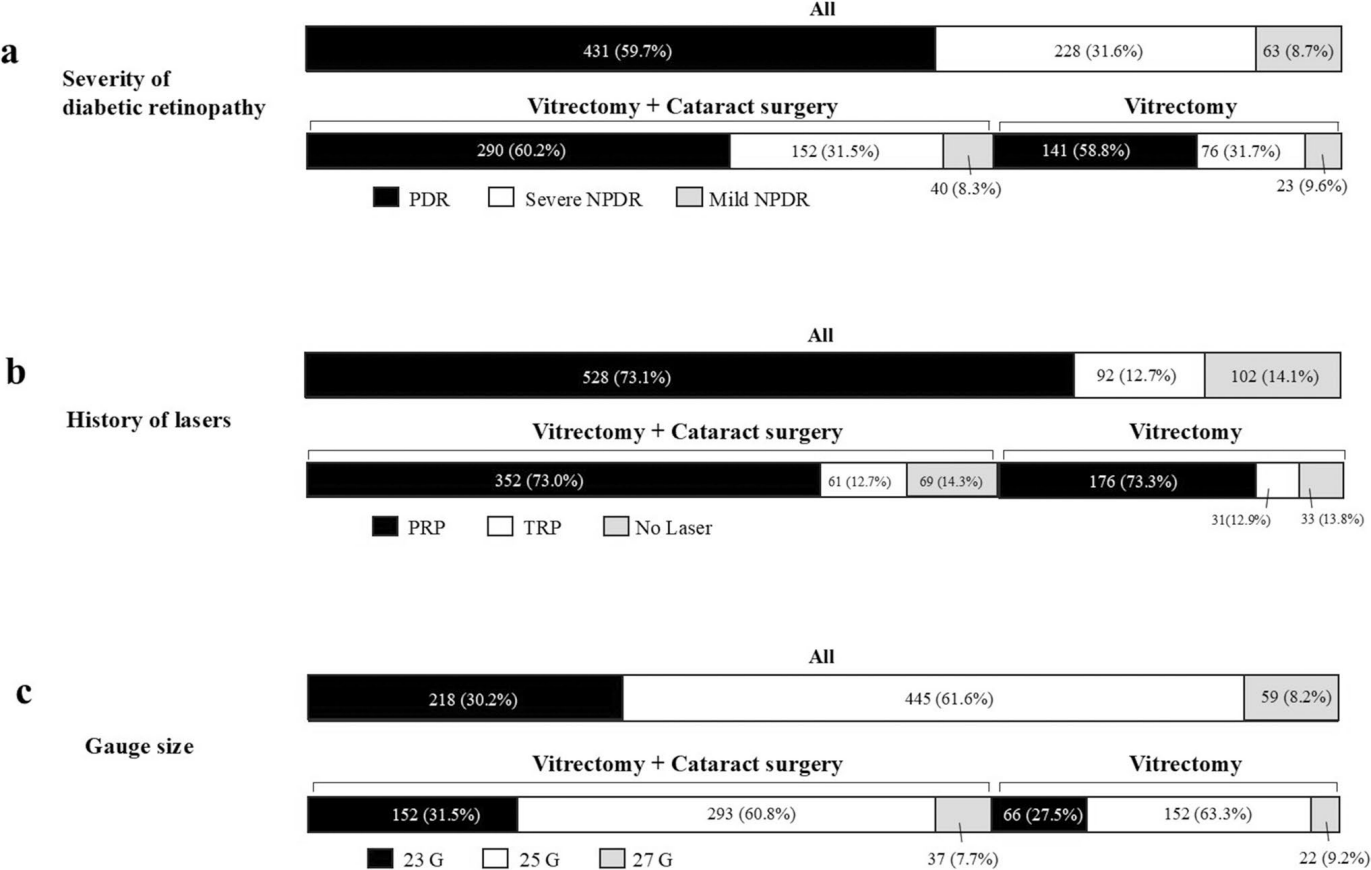
**a** Distribution of different grades of diabetic retinopathy, **b** the pattern of lasers in past history, and (**c**) gauge size employed during vitrectomy across all eyes, as well as in the eyes that underwent both vitrectomy and cataract surgery (VIT + CS group) and those that underwent only vitrectomy (VIT group)

Between 2010 and 2024, 722 patients who met the inclusion criteria were enrolled at 22 facilities in Japan. The baseline characteristics of the patients are presented in Table 1. The numbers of the patients in the VIT + CS group and VIT group were 482 and 240, respectively. The cases of lens sparing vitrectomy were excluded, and thus the all eyes in the VIT group were pseudophakic. The age of VIT + CS group was 64.4 ± 8.5 (range 48 to 83), and that in the VIT group was 65.1 ± 10.1 (49 to 89). The duration between the date diagnosed as DME and vitrectomy was 5.63 ± 7.67 years and 4.79 ± 6.51 years in the VIT + CS group and the VIT group, respectively. The number of intravitreal injections of anti-VEGF agents performed for 1 year before surgery was 1.01 ± 1.58 and 1.26 ± 1.75 in the VIT + CS group and VIT group, respectively. No significant differences between the VIT and VIT + CS groups were observed in terms of age, sex, hemoglobin A1c level, serum creatinine level, rate of insulin therapy and hemodialysis, or number of laser spots. Figure 1 shows the distribution of the severity of DR, the history of lasers and the gauge size. The ratio of the patients with proliferative diabetic retinopathy (PDR), severe non-PDR (NPDR), and mild NPDR were 59.7%, 31.6%, and 8.7%, respectively. The rate of patients with a history of laser prior to vitrectomy was 85.8% (PRP 73.1%, TRP 12.7%).

**Fig. 2:**
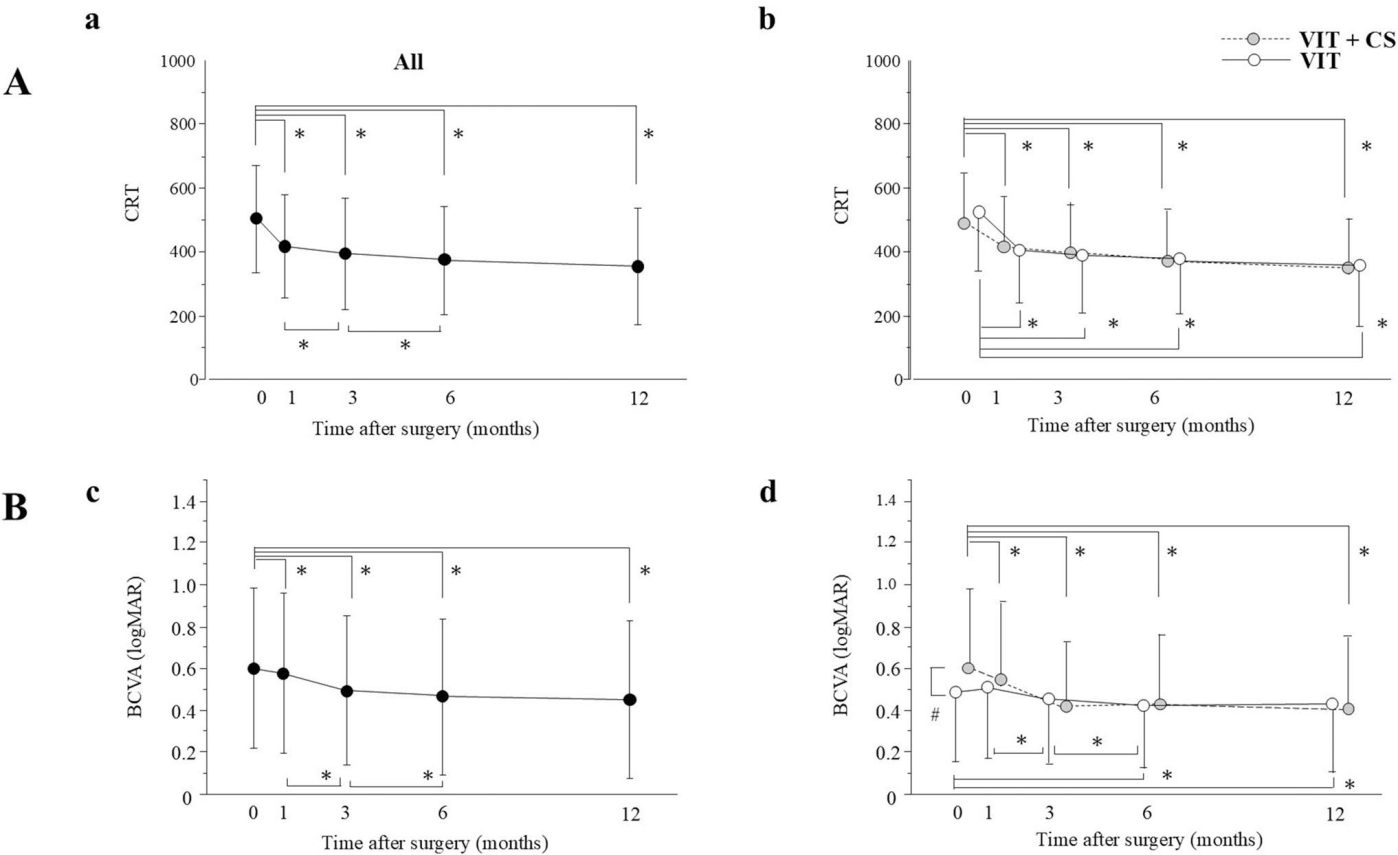
**A** Change in central retinal thickness (CRT) postoperatively for all eyes (**a**), as well as for the VIT group and the VIT + CS group (**b**). (**B**) Change in best corrected visual acuity (BCVA) postoperatively for all eyes (**c**) and for eyes that underwent only vitrectomy (VIT group) versus those that underwent vitrectomy with cataract surgery (VIT + CS group) (**d**). ^*^p < 0.05 (CRT or BCVA compared with baseline). ^#^*p* < 0.05 (VIT group vs. VIT + CS group)

In all eyes, CRT significantly improved at 1 month and thereafter (*p* < 0.0001) compared to baseline. A significant reduction in CRT was observed at each time point in the VIT + CS and VIT groups (Fig. 2A). In all eyes, BCVA significantly improved at 1 month (*p* = 0.0186) and thereafter (*p* < 0.0001) compared to baseline. Significant improvements were observed between 1 and 3 months, as well as between 3 and 6 months (*p* < 0.0001). In the VIT + CS group, the change in BCVA was significant at each time point compared to preoperative values (*p* < 0.0001). In contrast, in the VIT group, a significant improvement in BCVA was observed at 6 and 12 months postoperatively (*p* < 0.0001), with significant improvements also occurring between 1 and 3 months and between 3 and 6 months postoperatively (*p* < 0.0001).

**Fig. 3:**
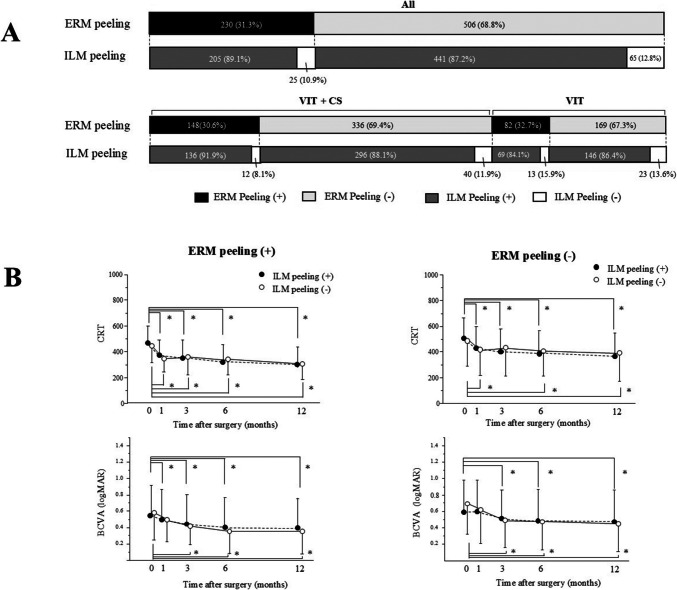
**A** Distribution of the cases involving the removal of epiretinal membrane (ERM) and internal limiting membrane (ILM) peeling. The black, light gray, dark gray, and white bars represent the presence of ERM peeling, absence of ERM peeling, presence of ILM peeling, and absence of ILM peeling, respectively. **B** Temporal profiles of CRT and BCVA in relation to the presence or absence of ERM peeling and ILM peeling. ^*^p < 0.05 (CRT or BCVA compared with baseline)

ERM was removed in 31.3%, 30.6%, and 32.7% of all patients, the VIT + CS group, and the VIT group, respectively (Fig. 3A). ILM peeling was performed in 91.9% and 88.1% of patients in the groups with and without ERM removal, respectively. Figure 3B shows that the CRT and BCVA were significantly improved in patients who underwent ERM peeling, both with and without ILM peeling. Similarly, in the group without ERM peeling, the CRT and visual acuity improved regardless of ILM peeling. No significant differences in CRT or BCVA were observed at any time point between patients with and without ILM peeling. The ILM staining reagents used included brilliant blue G (91.2%), indocyanine green (5.3%), and triamcinolone acetonide (3.4%).

**Fig. 4:**
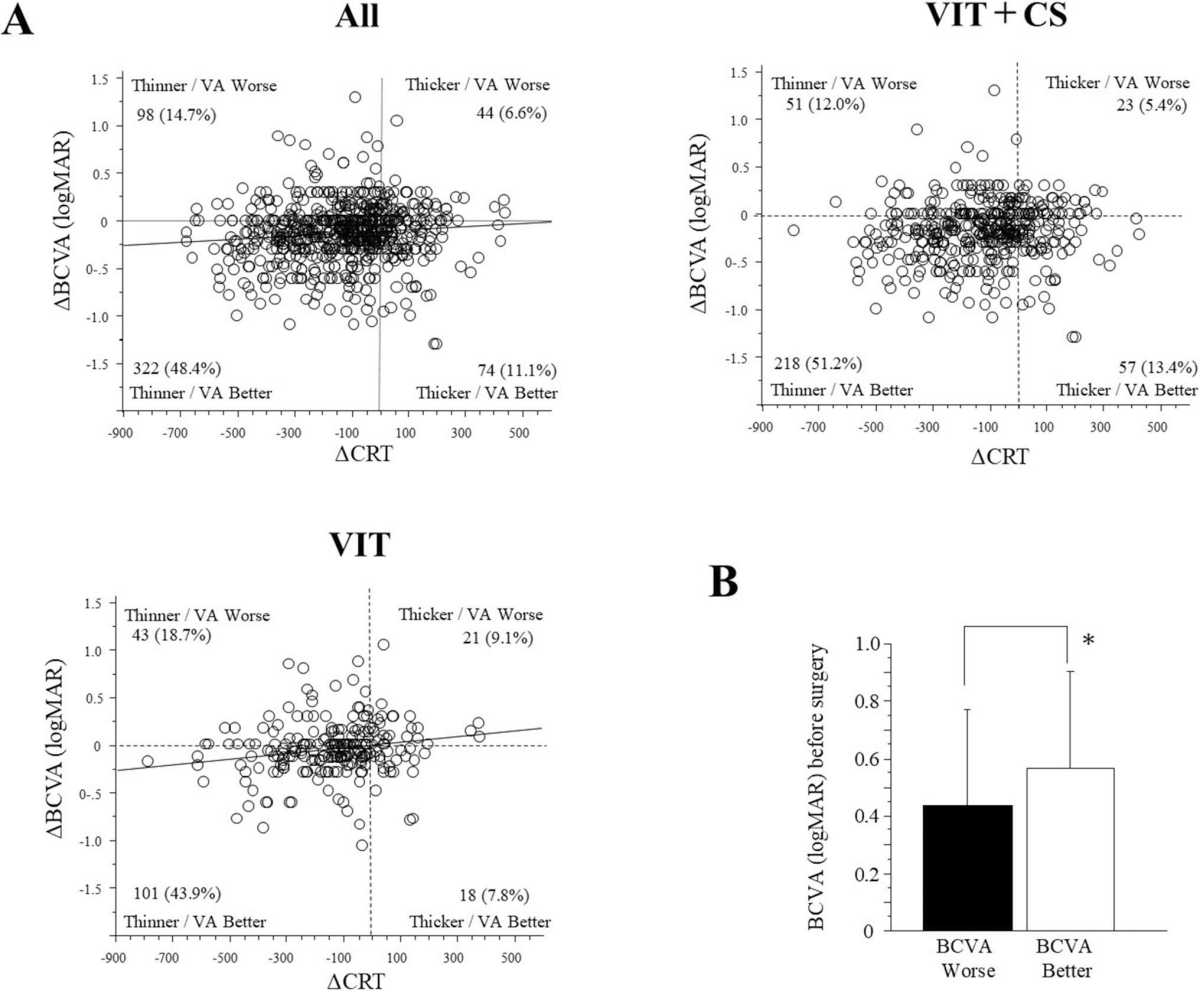
**A** Linear correlation between the change of central CRT and BCVA from baseline to 6 months after surgery. The solid line represents the regression line. **B** Comparison of preoperative BCVA between groups with worsening BCVA postoperatively and those with improving BCVA. ^*^p < 0.05

Figure 4A demonstrates a significant correlation between changes in CRT from baseline to 6 months and changes in BCVA for all patients (*p* = 0.0086; R^2^ = 0.011) and in the VIT group (*p* = 0.0205. R^2^ = 0.023). However, this correlation was not significant in the VIT + CS group. In the VIT group, unaffected by cataract surgery, we compared those with reduced CRT and improved vision to those with reduced CRT but worsened vision. Factors analyzed included age, sex, ERM removal, ILM peeling, HbA1c level, serum creatinine level, CRT, and BCVA at surgery. A significant difference was observed only in the preoperative BCVA, where the group with improved visual acuity had a significantly worse preoperative BCVA than the group with worsened visual acuity (*p* = 0.0317; Fig. 4B).

**Fig. 5:**
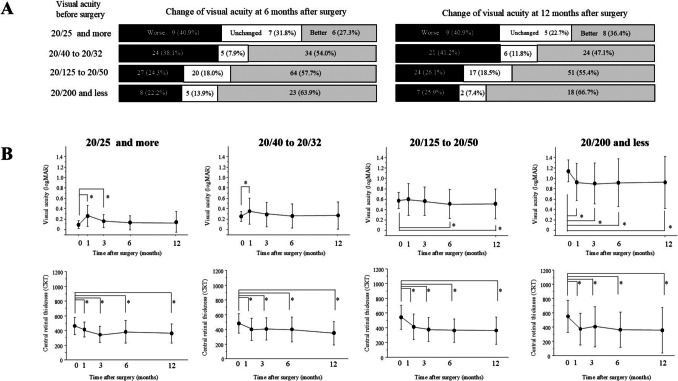
**A** Distribution of the change in visual outcome based on different preoperative BCVA. The black, white, and light gray bars represent worse, unchanged, and better changes of BCVA. **B** Temporal profiles of BCVA and CRT according to different preoperative BCVA. ^*^p < 0.05 (CRT or BCVA compared with baseline)

We categorized the patients into four groups based on preoperative BCVA: better VA (20/25 or more), relatively better VA (20/40 to 20/32), relatively worse VA (20/125 to 20/50), and worse VA (20/200 or less). We assessed visual acuity improvement in each group at 6 and 12 months post-surgery. Patients with worse preoperative visual acuity had a higher percentage of postoperative improvements (Fig. 5A). In the better and relatively better VA groups, BCVA transiently worsened at 1 month postoperatively and then improved, with no significant difference from the preoperative level (Fig. 5B). In contrast, the relatively worse VA group experienced significant BCVA improvements at 6 and 12 months postoperatively, while the worse VA group showed improvements starting at one month and continuing thereafter. In all groups, CRT improved significantly at 1 month postoperatively and beyond.

**Fig. 6:**
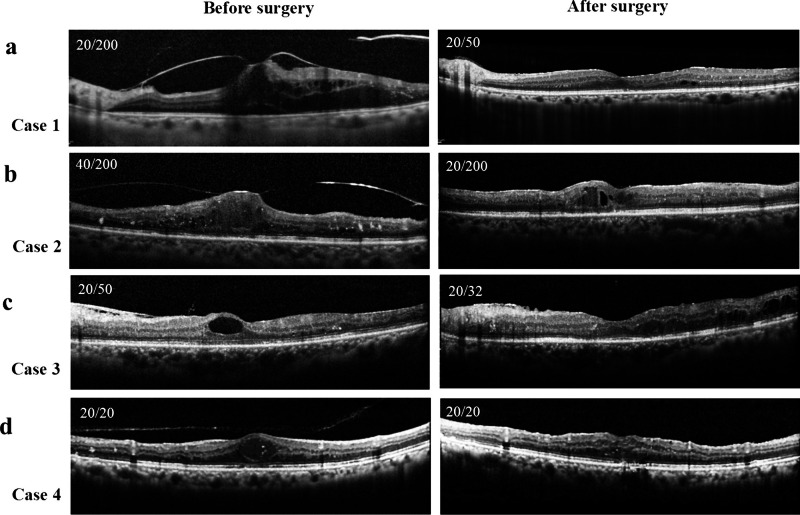
Representative cases show the change of OCT images between before and at 6 months after vitrectomy (Case 1: 49-year-old male, case 2: 65-year-old female, case 3: 59-year-old female, case 4: 67-year-old male). The BCVA improved postoperatively in case 1 and 3, worse in case 2, and unchanged in case 4. The values shown in upper left corner in each image are best corrected visual acuity

Figure 6 shows the OCT finding of 4 representative cases in which the vitrectomy was performed. In case 1 and 2, the vitreomacular traction was released by the vitrectomy. In case 1, the BCVA improved from 20/200 to 20/50. On the other hand, in case 2, intraretinal fluid was remained and the BCVA decreased from 40/200 to 20/200. In case 3, the cyst in the foveal area disappeared after vitrectomy, and the BCVA improved from 20/50 to 20/32. In case 4, the vitreous attachment was released, edema was absorbed, and the good visual acuity (20/20) was maintained postoperatively.

**Fig. 7:**
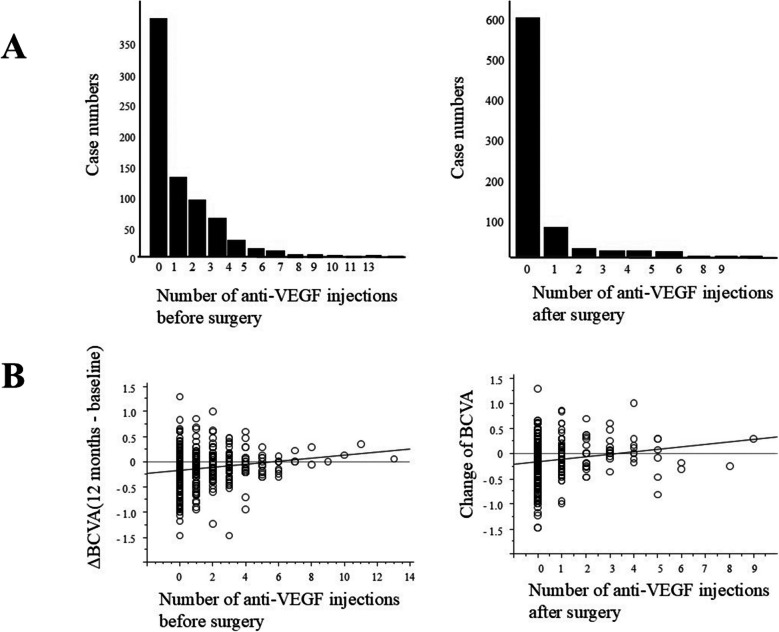
**A** The number of cases that received intravitreal injections of anti-VEGF agents before and after surgery for 1 year. **B** Linear correlation between the change of BCVA for 12 months after surgery and the number of injections of anti-VEGF agents for 1 year preoperatively and postoperatively. The solid line represents the regression line

The average number of anti-VEGF injections per 1 year before surgery was 1.08 ± 1.64, while after surgery it decreased to 0.32 ± 0.99. Both preoperatively (*P* < 0.0001, R^2^ = 0.025) and postoperatively (*P* = 0.0002, R^2^ = 0.023), the greater the number of injections correlated with worse the visual acuity at 1 year after surgery (Fig. 7). Regarding postoperative complications, NVG developed in 1.93% (14/722) of patients within 12 months, with 12 of these patients experiencing worse vision at 12 months compared to their preoperative status. Vitreous hemorrhage occurred in four patients, one of whom underwent additional vitrectomy.

## Discussion

This retrospective multicenter study demonstrated that CRT and BCVA significantly improved after vitrectomy for DME. Vitrectomy alone improved CRT and visual acuity, suggesting the effectiveness of vitrectomy for DME, consistent with previous reports [5, 6, 10]. In Japan, cataract surgery is performed simultaneously in combination with vitrectomy when cataracts are complicated. No significant difference in CRT changes was observed between the VIT + CS and VIT groups at any time point, and both groups rapidly improved significantly 1 month after surgery. In contrast, the BCVA in the VIT + CS group improved rapidly at 1 month after surgery, whereas it took 3 months in the VIT group. This rapid visual improvement in the VIT + CS group may be due to the removal of lens opacity. Our data suggest that vitrectomy, with and without cataract surgery, can lead to anatomical and functional improvements in patients with DME with cataracts.

After monotherapy with cataract surgery, the worsening of DME and consequent vision loss are frequently observed [12, 13], which is attributed to increased VEGF levels [14]. Intravitreal injections of anti-VEGF drugs at the end of cataract surgery in patients with DME have been reported to prevent postoperative DME worsening and may even improve it [12]. In this study, DME in the VIT + CS group improved at 1 month after surgery, likely due to VEGF and inflammatory cytokines washout from vitrectomy and increased clearance of chemical mediators [8, 15].

BCVA improved from 1 to 3 months and from 3 to 6 months postoperatively, suggesting that visual acuity continues to improve slowly after vitrectomy for DME. Unlike anti-VEGF therapy, where visual improvement occurs early after treatment, vitrectomy leads to a slower, more prolonged recovery.

Vitrectomy with ERM is often chosen for treating DME. Lewis et al. reported that vitrectomy is effective when associated with thick posterior vitreous membranes [6]. In the present study, significant improvements in CRT and visual acuity were observed in patients both with and without ERM, consistent with previous studies [5, 18]. These findings indicate that vitrectomy is effective in DME regardless of ERM presence.

Several reports have shown that ILM peeling during vitrectomy for DME results in more favorable anatomical and visual outcomes and a lower ERM recurrence rate [9, 19]. In contrast, it was reported that no significant change in CRT or visual acuity was observed in the eyes treated with ILM peeling [20]. Based on our report, ILM peeling did not significantly affect visual acuity or CRT improvement, regardless of ERM presence. The need for routine ILM peeling during vitrectomy in DME remains controversial.

 In the VIT group, changes in visual acuity and CRT at 6 months were correlated, indicating that as edema improved, so did visual acuity. This correlation was not significant in the VIT + CS group, possibly due to vision improvement from lens opacity removal. DRCR. net Protocol D also reported that CRT decreased after vitrectomy in more than 80% of eyes with vitreomacular traction, with 28–49% improving in visual acuity and 13–31% showing worsened acuity. Similarly, our data showed CRT reduction in 79.2% of eyes, with 43.9% improving in vision and 18.7% worsening despite CRT improvement in the VIT group. Since lens-sparing vitrectomy was performed using the DRCR.net, postoperative vision loss could have been caused by cataract progression [10]. In our study, lens-sparing vitrectomy was excluded and cataract surgery was combined with vitrectomy, which means that the cause of postoperative vision loss was other than cataract progression.

Characterizing cases where vision worsens despite CRT improvements is clinically important for evaluating the significance of vitrectomy. We found that patients with better preoperative BCVA had a higher likelihood of decreased visual acuity at 6 and 12 months post-surgery. In contrast, CRT improved regardless of preoperative visual acuity. This dissociation suggests that anatomical improvement of the macula is insufficient for functional recovery and further vision improvement.

Patients with good preoperative BCVA experienced a temporary decline in visual acuity at 1 month postoperatively, likely due to surgery-induced inflammation and neuronal tissue involvement. For those with BCVA of 20/40 or better, visual acuity at 6 and 12 months post-surgery was not significantly different from preoperative levels. Statistically, good preoperative vision was maintained after vitrectomy, however approximately 40% of these patients showed worsening in postoperative vision. This indicates that the vitrectomy is not a valid option to maintain good vision unless there are some threatening conditions such as vitreomacular traction impending foveal area. Instead, the choice of repeated intravitreal pharmacotherapy should be considered. In contrast, patients with poor preoperative BCVA showed a rapid improvement in both CRT and BCVA, highlighting vitrectomy's potential to enhance vision in cases with visual acuity below 20/50.

Anti-VEGF therapy, which is less invasive than vitrectomy and offers rapid improvements in CRT and vision, is the first-line treatment for DME. In this study, 7.5% of patients had a history of anti-VEGF drug injections four or more times per year before surgery. The lack of edema improvement despite frequent anti-VEGF administration may be the basis for the surgeon's decision to perform a vitrectomy. However, our data indicate that even with vitrectomy, visual acuity may decline, suggesting that vitrectomy may be less effective in patients resistant to anti-VEGF treatment.

DME can occur at any stage of diabetic retinopathy [21]. In this case series of patients who underwent vitrectomy, approximately 90% had PDR or severe NPDR, and 73.1% had a history of PRP, indicating the progressed retinal ischemia in many cases. Anti-VEGF therapy is the first choice for DME, while it is suggested that vitrectomy was selectively introduced in cases of DME associated with advanced DR. NVG occurs more frequently in patients with advanced retinal ischemia [22]. In our study, NVG occurred in about 2% of cases, resulting in postoperative vision loss. To prevent vitreous hemorrhage and NVG postoperatively, it is crucial to implement measures such as adequate intraoperative laser treatment and anti-VEGF therapy.

A limitation of this study is the potential variability in treatment outcomes due to differences in surgeon skills. Additionally, the morphological evaluation was limited to CRT using OCT, preventing a detailed analysis of the structural changes in the OCT images, such as subretinal detachment and intraretinal fluid. These parameters may be biomarkers for good or poor outcome after vitrectomy. The data based on fluorescein angiography and OCT-angiography were insufficient in this study, however the analysis regarding macular perfusion would also be important. This study is retrospective and consequently limited in drawing definitive conclusions, and the cohort includes only Japanese patients, so the results are not completely generalizable.

In conclusion, we demonstrated that vitrectomy effectively improves both the structural and functional aspects of DME, regardless of ERM presence. It helps maintain good vision in patients with favorable preoperative visual acuity and serves as a beneficial option for enhancing visual acuity in those with poor preoperative vision of 20/50 or worse. However, approximately 25% of patients with good preoperative visual acuity and 40% with poor preoperative visual acuity experienced worsening BCVA postoperatively. Therefore, the decision to perform vitrectomy should involve careful consideration of the associated risks and potential benefits.

## Data Availability

The datasets generated and/or analyzed in the current study are available from the corresponding author upon reasonable request.

## Appendix

The investigators and participants of the J-CREST (Japan Clinical REtina STudy group) and their affiliations: Yutaka Yamada^1^, Yoshihiro Takamura^1^, Masakazu Morioka^1^, Makoto Gozawa^1^, Masaru Inatani^1^, Kazuma Saito^2^, Akira Minamoto^3^, Gaku Ishigooka^4^, Takashi Koto^5^, Yuki Nakano, Eiko Tsuiki^7^, Hiroto Terasaki^8^, Miho Shimizu^9^, Masayo Kimura^10^, Yoshinori Mitamura^11^, Reio Sekine^12^, Yutaro Mizusawa^13^, Takao Hirano^14^, Tomoyuki Oyama^15^, Takeshi Iwase^16^, Fumiaki Higashijima^17^, Hisashi Matsubara^18^, Shigeo Yoshida^19^, Masahiko Sugimoto^20^, Sentaro Kusuhara^21^, Junya Hanaguri^22^.

^1^ Department of Ophthalmology, Faculty of Medical Sciences, University of Fukui, Yoshida, Japan.

^2^ Department of Ophthalmology, Gunma University Graduate School of Medicine, Maebashi, Japan.

^3^ Department of Ophthalmology and Visual Science, Hiroshima University, Hiroshima, Japan.

^4^ Department of Ophthalmology, Osaka Medical and Pharmaceutical University, Takatsuki, Japan

^5^ Department of Ophthalmology, Kyorin University School of Medicine, Tokyo, Japan.

^6^ Department of Ophthalmology, Kagawa University Faculty of Medicine, Miki, Japan.

^7^ Department of Ophthalmology and Visual Sciences, Graduate School of Biomedical Sciences, Nagasaki University, Nagasaki, Japan.

^8^ Department of Ophthalmology, Kagoshima University Graduate School of Medical and Dental Sciences, Kagoshima, Japan.

^9^ Sapporo City General Hospital Ophthalmology, Hokkaido, Japan.

^10^ Department of Ophthalmology and Visual Science, Nagoya City University Graduate School of Medical Sciences, Nagoya, Japan.

^11^ Department of Ophthalmology, Tokushima University Graduate School, Tokushima, Japan.

^12^ Department of Ophthalmology, St Marianna University School of Medicine, Kawasaki, Japan.

^13^ Department of Ophthalmology, Nara Medical University, Kashihara, Japan.

^14^ Department of Ophthalmology, Shinshu University School of Medicine, Matsumoto, Japan.

^15^ Department of Ophthalmology, National Defense Medical College, Tokorozawa, Japan.

^16^ Department of Ophthalmology, Akita University Graduate School of Medicine, Akita, Japan.

^17^ Department of Ophthalmology, Yamaguchi university school of medicine, Ube, Japan.

^18^ Department of Ophthalmology, Mie University Graduate School of Medicine, Tsu, Japan.

^19^ Department of Ophthalmology, Kurume University School of Medicine, Kurume, Japan.

^20^ Department of Ophthalmology, Yamagata University, Yamagata, Japan.

^21^ Division of Ophthalmology, Department of Surgery, Kobe University Graduate School of Medicine, Kobe, Japan.

^22^ Department of Ophthalmology, Nihon University Itabashi Hospital, Japan.
